# Development and Evaluation of an AI-Powered MRCPsych CASC Simulator for Exam Preparation

**DOI:** 10.1192/bjo.2025.10127

**Published:** 2025-06-20

**Authors:** Sirous Golchinheydari

**Affiliations:** West London NHS Trust, London, United Kingdom

## Abstract

**Aims:** Preparation for the MRCPsych CASC exam can present unique challenges for psychiatry trainees, including limited access to structured practice, real-time feedback and standardized patient interactions. This project aimed to develop the MRCPsych CASC Simulator (MCS), a custom AI-powered tool designed to enhance exam preparation by providing interactive clinical simulations, structured feedback and objective performance assessment.

**Methods:** The simulator incorporated three core roles – Doctor (candidate), Patient (actor), and Examiner – to create realistic CASC exam stations. MCS was trained in the functional aspects of the CASC, the requirements of both doctor and patient roles, along with the psychiatric expertise, knowledge and resources required. To test performance, we utilized validated assessment tools, including the examiner’s marking sheet for the CASC, Simulated Patient Rating Scale (SPRS), Objective Structured Clinical Examination (OSCE) the Communication Assessment Tool (CAT) to ensure objective and standardized evaluation. The simulator was tested in two roles, doctor and patient, by two different human assessors. The interactions were recorded and replayed for each assessment. Five stations were completed for each role from various psychiatric specialties. These scores were used to compare MCS with stock ChatGPT and to gain an overall understanding of MCS’ performance. Additionally, assessors requested MCS for immediate feedback on their questioning style, response phrasing, diagnostic accuracy and communication skills to gauge MCS’ effectiveness in providing feedback.

**Results:** The assessors found that MCS was competent in psychiatric assessments and patient simulation. MCS provided comprehensive learning support including mnemonics, diagnostic frameworks and summaries which facilitated differential diagnosis, clinical reasoning and memorisation. MCS provided real-time performance tracking, allowing potential candidates to refine their skills through iterative practice and targeted improvements.

MCS proved to be a significantly more effective tool for CASC practice than stock ChatGPT, scoring higher in both doctor and patient roles. MCS outperformed stock ChatGPT by an average 58% in doctor roles and 25% better in patient roles. Overall, the assessors found MCS to be a vital tool in CASC preparation.

**Conclusion:** MCS offers a novel and effective approach to psychiatric exam training by providing structured, objective and interactive practice opportunities. Its ability to provide tutoring, simulate realistic patient interactions and offer personalized feedback enhances clinical reasoning, communication skills and exam preparation.

